# Treatment of Acute Liver Failure in Resource-Constrained Settings without Transplantation Facilities Can Be Improved

**DOI:** 10.3389/fmed.2016.00031

**Published:** 2016-07-08

**Authors:** Francesca Cainelli, Bruno Nardo, Dmitriy Viderman, Bartholomew Dzudzor, Kenneth Tachi, Sandro Vento

**Affiliations:** ^1^Department of Medicine, School of Medicine, Nazarbayev University, Astana, Kazakhstan; ^2^Department of Medical and Surgical Sciences, S. Orsola-Malpighi Hospital, University of Bologna, Bologna, Italy; ^3^Department of Biomedical Sciences, School of Medicine, Nazarbayev University, Astana, Kazakhstan; ^4^Department of Medical Biochemistry, College of Health Sciences, School of Biomedical and Allied Health Sciences, University of Ghana, Accra, Ghana; ^5^Department of Medicine and Therapeutics, College of Health Sciences, School of Medicine and Dentistry, University of Ghana, Accra, Ghana

**Keywords:** acute liver failure, developing countries, intensive care, portal vein arterialization, treatment

Acute liver failure affects previously healthy and often young people and has a very high mortality due to rapid multi-organ failure. The diagnosis is based on the presence of coagulopathy (International normalized ratio >2 or prothrombin rate <50%) and hepatic encephalopathy within 8 weeks of onset of jaundice in patients with no previous liver disease (1).

In resource-constrained developing countries, hepatitis A, B, and E infections, traditional medicines (2), drugs, herbal supplements (3), and halothane (4) are the main causes. Even though liver transplantation is the treatment of choice nowadays, the survival rate without liver transplantation in adults is as high as 40% in high-income countries (5, 6). Unfortunately, it is much lower in developing countries (7) where cases are diagnosed too late or considered untreatable in the absence of a transplant center.

We propose a practical approach that can be used in Intensive Care Unit-equipped hospitals in resource-constrained countries to try and reduce the high mortality rate where liver transplantation is not available.

Patients admitted to general medical wards with signs or symptoms of acute liver failure should be screened for acute A, B, E, and possibly D virus hepatitis infections, for anti-nuclear and anti-smooth muscle autoantibodies (when possible) and for recent acetaminophen use, and prothrombin time should be immediately determined. Infections (typhoid fever, leptospirosis, rickettsial fever, falciparum malaria, and tuberculosis), which may mimic the presentation of acute liver failure should be excluded; high grade fever, splenomegaly, normal international normalized ratio, mild alanine aminotransferase and aspartate aminotransferase elevation help to exclude acute liver failure (8). As bacterial infections are associated with increased death rates (9), blood and urine cultures should be routinely checked at admission in all patients with acute liver failure. Good infection control measures are mandatory to reduce the risk of nosocomial sepsis.

Patients initially fully oriented can progress in few hours to acute delirium with agitation and then coma with response to painful stimuli only; thus, they should be assessed as soon as possible after admission using the West Haven Criteria (10), which have four grades: Grade I – lack of awareness, inverted sleep pattern, euphoria or anxiety, and shortened attention span; Grade II – lethargy or apathy, minimal disorientation for time or place, subtle personality change, and inappropriate behavior; Grade III – somnolence to semi stupor but responsive to verbal stimuli, confusion, and gross disorientation; and Grade IV – coma.

Patients with Grade I or II encephalopathy should be intubated and ventilated in case of sudden deterioration in consciousness level and transferred to the Intensive Care Unit. If not intubated, attention must be paid to aggressive behavior, hyperreflexia, and ankle clonus as they predict progression to Grade III–IV coma and development of severe brain edema (11). Appropriate intensive care can reduce the incidence of severe encephalopathy and intracranial hypertension, which develops more frequently in younger patients (whose skull is tighter), in patients with renal dysfunction and in those requiring inotropic support for cardiac failure (12). The patients should be kept in a 20°–30° head-up position to decrease intracranial pressure and reduce the risk of aspiration pneumonia. Fluid should be restricted to two-third maintenance and glucose levels maintained at >4.0 μmol/l; gastroprotective drugs (e.g., ranitidine and sucralfate) should be used for prevention of gastrointestinal bleeding, and sepsis should be prevented with broad spectrum antibiotics. Large-volume infusions of hypotonic fluids, which may result in hyponatremia and cerebral swelling, should be avoided. Due to the high protein catabolism, 1.0–1.5 g of enteral protein/kg/day can be given with blood ammonia levels monitoring.

Antifungal therapy should be started in patients who do not improve with antibiotics. Fever may favor development of cerebral edema, by increasing body metabolism and production of ammonia, and its cerebral uptake and metabolism. Body temperature should be kept at 35–36°C using external cooling and suppressing shivering.

Routine computerized tomography scan to detect brain edema should not be performed as moving the patient to the scanner might facilitate increase in intracranial pressure; computerized tomography scan (where available) should be done only when focal neurological signs or pupillary abnormalities (suggesting intracranial bleeding or cerebral herniation) are present. Cerebral edema can be diagnosed clinically based on the presence of spontaneous decerebrate posturing alone or two of the following: hypertension, bradycardia, pupillary changes, neurogenic hyperventilation (13). For clinical signs of increased intracranial pressure, a bolus of intravenous hypertonic saline (at a dose of 20 ml of 30% sodium chloride, keeping serum sodium at <150 mmol/l) or mannitol (2 ml of 20% solution/kg b.w.) should be used.

Correction of coagulopathy is indicated only before an invasive procedure (e.g., insertion of a central line); indeed, the risk of hemorrhage correlates more with the severity of thrombocytopenia rather than coagulopathy. The presence of disseminated intravascular coagulation usually indicates sepsis, which must be considered also in case of unexplained blood pressure decrease, urinary output drop, worsening encephalopathy, and development of severe acidosis.

It is very important to anticipate and prevent renal failure by maintaining circulating volume with colloid or fresh frozen plasma (if available). Extracorporeal renal support in the form of hemofiltration may be necessary (when available), if there is an acute renal failure or concern about fluid management.

As for etiology-specific therapies, *N*-acetyl-cysteine administration is the treatment of choice for acetaminophen-related acute liver failure and can be effective also in non-acetaminophen acute liver failure with low grade encephalopathy (14). Nucleoside analogs can be employed in hepatitis B virus-related and corticosteroids in severe autoimmune hepatitis-related acute liver failure but are of dubious influence on prognosis (15).

A procedure that could be investigated as an additional treatment for acute liver failure is temporary partial portal vein arterialization [reviewed in Ref. (16)], which can be performed through mesenteric vessel anastomosis or an extracorporeal device (that withdraws blood from the femoral artery and return it in the portal venous system through the umbilical vein). The procedure could stimulate hepatocyte regeneration by supplying extra oxygen to the liver to match the increased metabolic demand of the regenerating cells and by increasing portal pressure (which can activate regeneration) (17). The success in animal models (18, 19) and in some acute liver failure cases (16) indicates that this simple-to-perform procedure merits further investigation in patients with acute liver failure and might be successful particularly in cases where this is due to drugs or toxics. Of course, it should be applied early enough in the course of the disease and when the patient does not meet, yet, the King’s College Hospital criteria for liver transplantation (20).

In conclusion, acute liver failure should not be considered untreatable without liver transplantation; chances of survival can be increased even in resource-constrained developing countries if patients are appropriately managed.

## Author Contributions

FC had the idea of writing the manuscript and drafted it. BN codrafted the manuscript. DV, BD, and KT contributed to the drafting. SV contributed to the drafting, corrected, and reviewed the manuscript. All the authors approved the final version.

## Conflict of Interest Statement

The authors declare that the research was conducted in the absence of any commercial or financial relationships that could be construed as a potential conflict of interest.
